# Leveraging Innate Immune and Metabolic Cell Death Pathways in Cancer Therapies

**DOI:** 10.34133/cancomm.0062

**Published:** 2026-11-12

**Authors:** Yaqiu Wang, Thirumala-Devi Kanneganti

**Affiliations:** Department of Immunology, St. Jude Children’s Research Hospital, Memphis, TN, USA.

## Abstract

Cancer cells evade cell death, and overcoming this resistance has been a fundamental challenge in cancer treatment. Innate immunity is critical in detecting cellular alterations, including those that accompany malignant transformation. Furthermore, innate immune signaling can activate tumor cell-intrinsic cell death pathways and immune-mediated killing to eliminate cancer cells. Several cell death pathways that are relevant to cancer have been defined, and we have broadly categorized these as caspase-dependent and caspase-independent pathways to reflect the key mechanistic difference between these groups for this discussion. The caspase-dependent pathways are genetically regulated by caspases; these include apoptosis, pyroptosis, and PANoptosis, which require caspases, and the contrasting pathway necroptosis, which is initiated in the absence of caspase-8. In contrast to caspase-dependent pathways, the caspase-independent pathways are driven by disruption of essential metabolic processes and include ferroptosis, disulfidptosis, cuproptosis, and mitoxyperilysis. Apoptosis is nonlytic, while all other pathways discussed here are lytic. Lytic cell death can involve the release of damage-associated molecular patterns (DAMPs) or cytokines that can further amplify innate immune cell death responses and paracrine PANoptosis, contributing to a sustained pro-inflammatory environment. Cancer cells vary widely in their expression of cell death mediators, leading to differing sensitivities to these cell death pathways. This heterogeneity represents both a therapeutic challenge and opportunity: inflammatory cell death within the tumor microenvironment is a double-edged sword, priming antitumor immunity in some contexts while fueling immunosuppression in others. In this review, we describe the cell death pathways relevant to cancer, highlighting recent advances in our mechanistic understanding of these pathways and discussing how this informs therapeutic targeting in cancers. Overall, a thorough understanding of innate immune responses in cancer cell death and the immunological consequences are essential to leverage these mechanisms to inform biomarker-guided patient stratification and develop next-generation cancer therapies.

## Introduction

Hallmarks of cancer include uncontrolled cellular proliferation and resistance to cell death [1]. Many conventional treatment strategies, apart from surgical tumor resection, aim to trigger cancer cell death directly (e.g., radiation and chemotherapy) or indirectly (e.g., immune checkpoint blockade-mediated activation of cytotoxic T cells that can kill cancer cells) [1–9]. Cell death pathways are critical components of the innate immune response and can be leveraged for anticancer therapy, with the potential to enhance adaptive antitumor immunity. Several key cell death pathways have clinical implications in cancers. Many of these pathways are regulated by caspases, with apoptosis, pyroptosis, and PANoptosis being driven by caspase activation, while necroptosis is triggered by caspase-8 inhibition [10]. Caspases have historically been recognized as master regulators of cell death, and they remain the most studied genetic regulators of cell death pathways. However, more recent advances in the cell death field have identified another category of cell death pathways, such as ferroptosis, disulfidptosis, cuproptosis, and mitoxyperilysis, which operate independently of caspases and are instead driven by disruption of metabolic processes. Most caspase-dependent and independent cell death pathways can be immunogenic, as they are lytic and release intracellular components known as damage-associated molecular patterns (DAMPs) to activate innate immune sensors to further amplify inflammatory cascades, cytokine release, and paracrine PANoptosis; the exception is apoptosis, which is nonlytic and often immunologically silent under homeostatic conditions. While the activation of cell death and inflammation is often beneficial for host defense, inflammation in the tumor microenvironment (TME) plays multifaceted roles in cancer progression, making context-specific targeting of these pathways critical [1,6]. Here, we provide a concise overview of key cell death pathways and discuss how they can be targeted in current and future anticancer therapies.

## Many Ways to Die—Cell Death Modalities in Cancer

Cell death is a central therapeutic target in cancers (Table 1). Harnessing it effectively requires a precise molecular understanding of the diverse pathways through which cells die (Table 2). Decades of research have defined the complexity of cell death regulation and highlighted the distinct contributions of each pathway to cancer biology.

**Table 1.** Representative existing and potential therapeutic strategies to induce cancer cell death

Therapeutic agent	Molecular target	Mechanism	Cancer type/models	Outcomes	Clinical status	References
Venetoclax	BCL-2	Sensitize cells to mitochondrial damage and apoptosis; BH3 mimetic	Mostly hematological malignancies	Approved to treat CLL, SLL, and AML	FDA approved	[94,95]
LCL161	IAP	Induction of apoptosis; SMAC mimetic	Myelofibrosis	30% overall response rate and notable toxicity	Phase II	[150]
Xevinapant with cisplatin chemoradiotherapy	IAP	Induction of apoptosis; SMAC mimetic	Head and neck cancer	Improved overall survival in phase II; worsened overall survival in phase III	Phase II/III	[151,152]
GSK3145095 ± pembrolizumab	RIPK1	Suppression of RIPK1-dependent inflammation and cell death pathways (e.g., necroptosis, PANoptosis, apoptosis); proposed to reprogram myeloid infiltrates and sensitize to anti-PD-1 treatment	Solid tumors	Terminated for unknown reasons	Phase I/II	[153]
6,7-Dichloro-2-methylsulfonyl-3-*N*-tert-butylaminoquinoxaline	GSDMD	Induction of lytic cell death (e.g., pyroptosis, PANoptosis); GSDMD cleavage-independent agonist	Various allograft mouse models	GSDMD-dependent tumor volume decrease	Preclinical	[106]
Decitabine + doxorubicin	N/A	Induction of RIPK3 expression and cell death in *RIPK3* hypermethylated cancer cells	Breast tumor xenograft mouse model	Decreased tumor volume	Preclinical	[37]
KPT-330 + IFN	N/A	Induction of ZBP1-dependent PANoptosis	B16 melanoma mouse model	Decreased tumor volume	Preclinical	[55]
TNF + IFN-γ	N/A	Induction of PANoptosis	Colon cancer xenograft mouse model	Decreased tumor volume	Preclinical	[56]
Sulfasalazine	SLC7A11	Induction of ferroptotic features; limit cystine uptake	Recurrent glioblastoma	Completed; compound safe and achieved 45% objective response rate	Phase I	[154]
Cyst(e)inase	N/A	Induction of ferroptosis; engineered enzyme to deplete cysteine/cystine, increase ROS, and deplete GSH	Multiple syngeneic and xenograft mouse models	Decreased tumor volume	Preclinical	[155]
Elesclomol + paclitaxel	N/A	Copper-dependent cytotoxic stress; mechanistically aligns with cuproptosis concept	Melanoma	No benefit to progression-free survival	Phase III	[132]
Cu_9_S_8_-based nanoplatform	N/A	Induction of cuproptosis	Breast cancer syngeneic mouse models	Suppressed development and metastasis	Preclinical	[131]
BAY-876	GLUT1	Induction of NADPH depletion and disulfidptosis in SLC7A11^high^ tumors	Lung cancer xenograft mouse models	Decreased tumor volume	Preclinical	[80]
LPS intratumoral injection with fasting	N/A	Induction of mitoxyperilysis	B16 melanoma mouse model	Decreased tumor volume	Preclinical	[86]

AML, acute myeloid leukemia; BCL-2, B cell lymphoma 2; BH3, BCL-2 homology 3, including BH3-only proteins; CLL, chronic lymphocytic leukemia; FDA, U.S. Food and Drug Administration; GLUT1, glucose transporter 1; GSDMD, gasdermin D; GSH, reduced glutathione; IAP, inhibitor of apoptosis protein; IFN, interferon; LPS, lipopolysaccharide; N/A, not available; NADPH, reduced nicotinamide adenine dinucleotide phosphate; PD-1, programmed cell death protein 1; RIPK, receptor-interacting serine/threonine-protein kinase; ROS, reactive oxygen species; SLC7A11, solute carrier family 7 member 11; SLL, small lymphocytic lymphoma; SMAC, second mitochondrial activator of caspase; TNF, tumor necrosis factor; ZBP1, Z-DNA-binding protein 1

**Table 2.** Key molecules involved in cell death pathways as potential therapeutic targets

Pathways of cell death	Key molecules involved	References
Apoptosis Nonlytic; caspases activated	Key molecules: P53, BH3-only and other BCL-2 family proteins, XIAP, cIAP1, cIAP2, CYTC, TRADD, FADD, RIPK1, APAF1, CASP8, CASP9, CASP3, CASP7	[11,13–16,20–28,156]
Necroptosis Lytic; CASP8 inhibition	Receptors: TNFR1, TLR3, TLR4, ZBP1 Regulators and cell death complex molecules: RIPK1, RIPK3 Executioners/pore-forming molecules: MLKL	[30–35,157]
Pyroptosis Lytic; innate immune; CASP1/4,5/11 activated; inflammasomes	Cytosolic innate immune sensors: AIM2, NLRC4, NLRP1, NLRP3, Pyrin, CASP4, CASP5 Cell death complex molecules: ASC, CASP1 Executioners/pore-forming molecules: GSDMD Immune effectors: IL-1β, IL-18	[43–47,158–171]
PANoptosis Lytic; Innate immune; caspases and RIPKs activated; PANoptosomes	Cytosolic innate immune sensors: AIM2, NLRC5, NLRP3, NLRP12, RIPK1, ZBP1 Regulators and cell death complex molecules: IRF1, FADD, RIPK1, RIPK3, ASC, CASP1, CASP6, CASP8, CASP3, CASP7 Executioners/pore forming molecules: GSDMD, GSDME, MLKL, others Immune effectors: IL-1β, IL-18, TNF, IL-6, other cytokines	[10,12,49–51,53–63]
Ferroptosis Lytic; iron-dependent lipid peroxidation	Key molecules: SLC7A11 (xCT), TFRC, GPX4, ACSL4, FSP1, LPCAT3	[69,71,72,75,172]
Disulfidptosis Lytic; disulfide stress	Key molecules: SLC7A11, RAC1, NCKAP1	[80]
Cuproptosis Lytic; copper-dependent	Key molecules: FDX1, DLAT, DLST, LIAS, LIPT1	[84]
Mitoxyperilysis Lytic; mTORC2-dependent	Receptors: TLRs, TNFR1 Key molecules: BAX, BAK, BID, RICTOR (mTORC2)	[86]

ACSL4, acyl-coenzyme A synthetase long-chain family member 4; AIM2, absent in melanoma 2; APAF1, apoptotic protease-activating factor 1; ASC, apoptosis-associated speck-like protein containing a caspase activation and recruitment domain; BAK, BCL-2 homologous antagonist/killer; BAX, BCL-2-associated X protein; BH3, BCL-2 homology 3, including BH3-only proteins; BID, BH3-interacting domain death agonist; CASP1, caspase-1; CASP3, caspase-3; CASP4, caspase-4; CASP5, caspase-5; CASP6, caspase-6; CASP7, caspase-7; CASP8, caspase-8; CASP9, caspase-9; cIAP1, cellular inhibitor of apoptosis protein 1; cIAP2, cellular inhibitor of apoptosis protein 2; CYTC, cytochrome c; DLAT, dihydrolipoamide S-acetyltransferase; DLST, dihydrolipoamide S-succinyltransferase; FADD, Fas-associated protein with death domain; FDX1, ferredoxin 1; FSP1, ferroptosis suppressor protein 1; GPX4, glutathione peroxidase 4; GSDM, gasdermin; IL-1β, interleukin-1β; IL-6, interleukin-6; IL-18, interleukin-18; IRF1, interferon regulatory factor 1; LIAS, lipoic acid synthetase; LIPT1, lipoyltransferase 1; LPCAT3, lysophosphatidylcholine acyltransferase 3; MLKL, mixed-lineage kinase domain-like pseudokinase; NCKAP1, NCK-associated protein 1; NLRC4, nucleotide-binding oligomerization domain-like receptor family caspase activation and recruitment domain-containing 4; NLRC5, nucleotide-binding oligomerization domain-like receptor family caspase activation and recruitment domain-containing 5; NLRP1, nucleotide-binding oligomerization domain-like receptor family pyrin domain-containing 1; NLRP3, nucleotide-binding oligomerization domain-like receptor family pyrin domain-containing 3; NLRP12, nucleotide-binding oligomerization domain-like receptor family pyrin domain-containing 12; P53, tumor protein p53; RAC1, Ras-related C3 botulinum toxin substrate 1; RICTOR, rapamycin-insensitive companion of mechanistic target of rapamycin; RIPK1, receptor-interacting serine/threonine-protein kinase 1; RIPK3, receptor-interacting serine/threonine-protein kinase 3; SLC7A11 (xCT), solute carrier family 7 member 11 (cystine/glutamate antiporter light chain); TFRC, transferrin receptor; TLR3, Toll-like receptor 3; TLR4, Toll-like receptor 4; TLRs, Toll-like receptors; TNF, tumor necrosis factor; TNFR1, tumor necrosis factor receptor 1; TRADD, tumor necrosis factor receptor 1-associated death domain protein; XIAP, X-linked inhibitor of apoptosis protein; ZBP1, Z-DNA-binding protein 1

### Cell death pathways involving caspases

The identification of caspase functions in cell death laid the foundational framework for understanding programmed cell death pathways [11]. The most well-studied cell death modalities are those dependent on caspase activation (apoptosis, pyroptosis, and PANoptosis) or inhibition (necroptosis) [12]. Apoptosis was the first regulated cell death pathway identified [13], and it proceeds through 2 distinct but mechanistically related initiation processes. Intrinsic apoptosis is initiated by mitochondrial damage, often mediated by BAX/BAK (BCL2-associated X protein/BCL-2 homologous killer) permeabilization and cytochrome c release to bind APAF1 (apoptotic protease-activating factor 1) in the cytosol and activate initiator caspase-9 [14]. In contrast, extrinsic apoptosis is initiated by death receptors, leading to the activation of initiator caspase-8 [15,16]. Despite these differences in initiation, both caspase-9 and caspase-8 go on to activate downstream effector caspase-3 and -7, which cleave their substrates [e.g., ICAD (inhibitor of caspase-activated DNase) for DNA degradation [17], ROCK1 (Rho-associated, coiled-coil-containing protein kinase 1) for bleb formation [18], and PARP1 (poly-[adenosine diphosphate-ribose] polymerase 1) to inactivate DNA repair [19]] to drive the apoptotic cell death (Fig. 1). Activated caspase-8 can also cleave BID (BH3 interacting domain death agonist), which induces mitochondrial damage to activate apoptosis execution through the intrinsic pathway [20]. p53 exerts influence on both intrinsic and extrinsic apoptosis through regulation of many apoptotic molecules, including DR5 (death receptor 5) [21], FAS (Fas cell surface death receptor) [22], BAX [23], BID [24], PUMA (p53 up-regulated modulator of apoptosis) [25], and NOXA (phorbol 12-myristate 13-acetate-induced protein 1) [26]. Both pathways can be inhibited by inhibitor of apoptosis (IAP) proteins, including cIAP1/2 (cellular inhibitors of apoptosis proteins 1/2) and XIAP (X-linked inhibitor of apoptosis), which are antagonized by SMAC/DIABLO (second mitochondria-derived activator of caspases) [27]. Although apoptosis was first identified in cancer cells, many cancer cells evade apoptosis through adaptations, such as up-regulating factors like BCL-2 (B cell lymphoma 2) and IAPs to antagonize apoptotic signaling, or down-regulating p53 and death receptors [1,28,29].

**Fig. 1.**
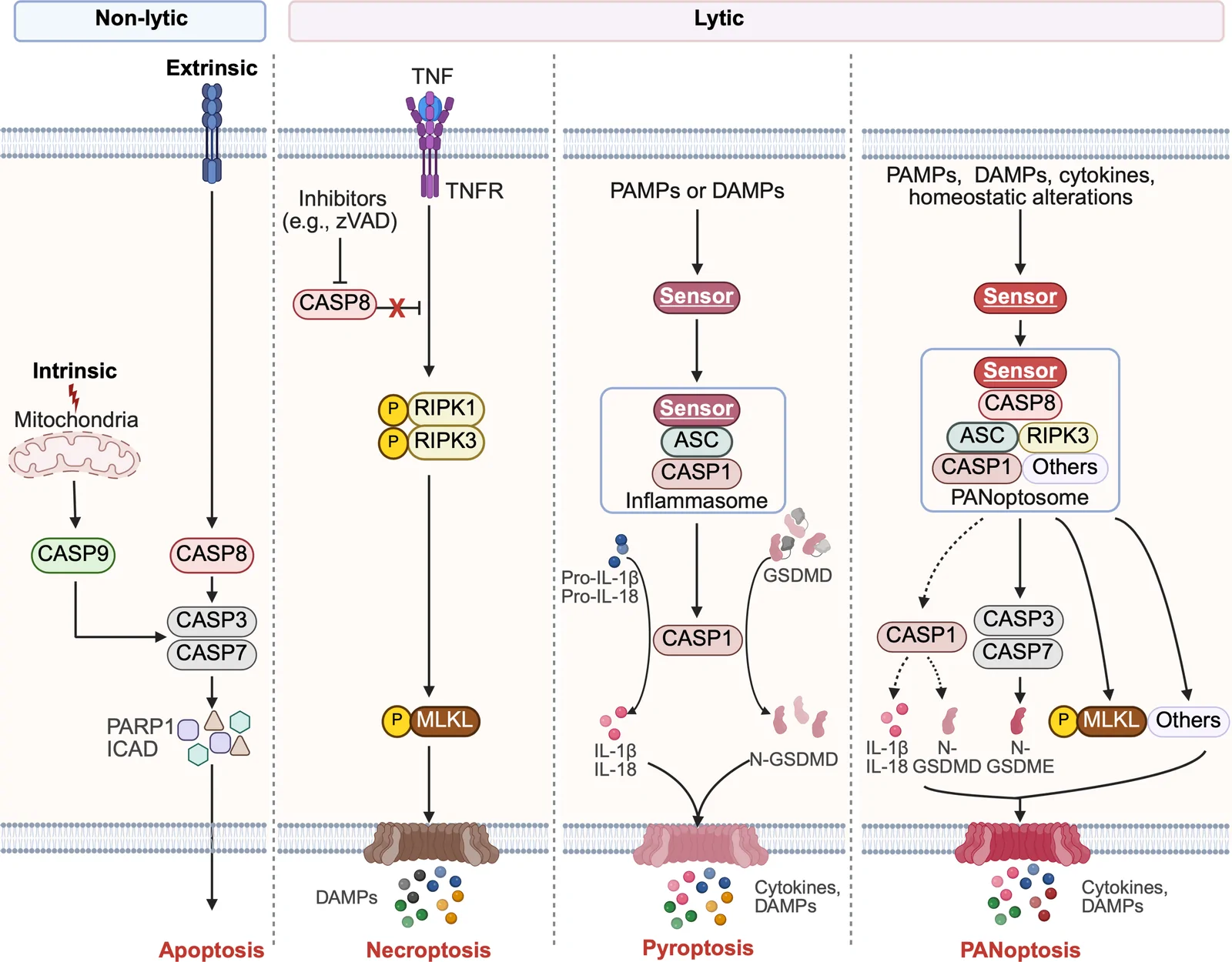
Cell death pathways involving caspases. Apoptosis is a nonlytic cell death pathway initiated through intrinsic [caspase-9 (CASP9)-dependent] and extrinsic [caspase-8 (CASP8)-dependent] routes and mediated by caspase-3 (CASP3) and caspase-7 (CASP7). Necroptosis, pyroptosis, and PANoptosis are lytic cell death pathways. Necroptosis is inhibited by caspase-8 and therefore requires its deletion or inhibition to be activated; it is driven by activated, phosphorylated RIPK1 and RIPK3, leading to MLKL phosphorylation (pMLKL) and membrane pore formation. Pyroptosis is initiated by innate immune sensors in response to pathogen- or damage-associated molecular patterns (PAMPs or DAMPs, respectively). The innate immune sensor forms an inflammasome complex with the adaptor ASC and the inflammatory caspase-1 (CASP1), leading to CASP1 activation and cleavage of its substrates gasdermin D (GSDMD) and the cytokines IL-1β and IL-18. The N-terminal fragment of GSDMD (N-GSDMD) can then form membrane pores to induce membrane lysis. PANoptosis is also initiated by innate immune sensors but involves the formation of a PANoptosome complex, containing caspase-8, as well as RIPKs and other adaptor and effector molecules. This complex formation leads to the activation of caspases and RIPKs, leading to membrane pore formation by gasdermins, MLKL, and other factors causing membrane lysis to release cytokines (IL-1β and IL-18) and DAMPs. This figure was generated using BioRender. ASC, apoptosis-associated speck-like protein containing a caspase activation and recruitment domain; CASP1, caspase-1; CASP3, caspase-3; CASP7, caspase-7; CASP8, caspase-8; CASP9, caspase-9; DAMPs, damage-associated molecular patterns; GSDMD, gasdermin D; ICAD, inhibitor of caspase-activated deoxyribonuclease; IL-1β, interleukin-1β; IL-18, interleukin-18; MLKL, mixed-lineage kinase domain-like pseudokinase; N-GSDMD, N-terminal fragment of gasdermin D; N-GSDME, N-terminal fragment of gasdermin E; P, phosphorylation; PAMPs, pathogen-associated molecular patterns; PARP1, poly(adenosine diphosphate-ribose) polymerase 1; pro-IL-1β, precursor interleukin-1β; pro-IL-18, precursor interleukin-18; RIPK1, receptor-interacting serine/threonine-protein kinase 1; RIPK3, receptor-interacting serine/threonine-protein kinase 3; TNF, tumor necrosis factor; TNFR, tumor necrosis factor receptor; zVAD, benzyloxycarbonyl-Val-Ala-Asp fluoromethyl ketone (a pan-caspase inhibitor).

In contrast to the activation of caspase-8 in the nonlytic apoptosis pathway, inhibition of caspase-8 leads to lytic cell death through necroptosis [30,31]. Necroptosis can also be driven by the loss of RIPK1 (receptor-interacting serine/threonine-protein kinase 1) scaffold function [32]. In both cases, this leads to RIPK3-mediated phosphorylation of the executioner MLKL (mixed lineage kinase domain-like pseudokinase) [33], which oligomerizes and inserts into the membrane to drive cell lysis (Fig. 1) [34–36]. The physiological function of necroptosis in cancer is context-dependent. Many cancer cells silence necroptosis by down-regulating its pathway mediators, such as RIPK3, suggesting an onco-suppressive role for necroptosis [37]. Additionally, in hepatocellular carcinoma (HCC), higher mRNA expression of *RIPK1*, *RIPK3,* and *MLKL* correlated with better overall survival and T cell infiltration [38], although whether such changes in expression reflect bona fide necroptosis activation or enhanced immune cell infiltration is unclear. In contrast, necroptosis was detrimental in a pancreatic cancer model involving *Kras*^G12D^ expression, where genetic deletion of RIPK3 was needed to alleviate immunosuppression in the TME and decrease tumor growth and metastasis [39]. A similar detrimental role for necroptosis was observed in a breast cancer model with mammary gland-specific expression of the polyomavirus middle T antigen, where necroptosis was also associated with tumor growth and metastasis [40]. Beyond these cancer-specific effects of necroptosis, cell death signaling can also influence tumor lineage specification. One such example is in liver cancer, where expression of the same oncogenic drivers induced development to either HCC or intrahepatic cholangiocarcinoma (ICC), depending upon the cell death signaling in the TME. Specifically, cell death signaling and cytokine profiles associated with necroptosis gave rise to ICC, while those associated with apoptosis gave rise to HCC, and RIPK1 inhibition shifts the development of ICC to HCC [41].

In contrast to apoptosis and necroptosis, pyroptosis and PANoptosis are activated by innate immune sensors that assemble caspase-containing inflammatory cell death complexes. Pyroptosis was initially identified through studies of the *Shigella*-induced cell death process. The role of caspase-1 was identified first [42], and this cell death pathway was later termed pyroptosis based on the involvement of inflammatory caspases and its pro-inflammatory nature associated with maturation cleavage of interleukin-1β (IL-1β) [43] and IL-18 [44] by caspase-1, which contrasts with that of apoptosis [45]. Mechanistically, pyroptosis is initiated by innate immune sensors that undergo activation and form inflammasome complexes, which induce the activation of caspase-1. Caspase-1 then cleaves the executioner protein GSDMD (gasdermin D), causing N-terminal GSDMD fragments (N-GSDMD) to form pores in the plasma membrane and drive cell lysis (Fig. 1); GSDMD can also be cleaved by caspase-4/5 (caspase-11 in mice) to drive this lysis [46–48].

PANoptosis is another form of inflammatory, lytic cell death initiated by innate immune sensors, and it is driven by caspases and RIPKs through PANoptosome complexes (Fig. 1) [10,12,49–63]. Inflammasomes can be integral components of PANoptosomes, along with caspase-8 and other core adaptor and effector molecules. PANoptosome formation leads to the activation of caspase-8, caspase-1, caspase-3, caspase-7, and RIPKs; these molecules then induce the activation of executioners such as GSDMD, GSDME (gasdermin E), MLKL, and others [12,49–51,53,56–63]. Ablation of upstream innate immune sensors or core machinery (e.g., caspase-8 together with RIPK3, or caspase-8 with RIPK3 and caspase-1) prevents PANoptosis [49,51]. Given the molecular overlap between proteins involved in PANoptosis and those involved in other cell death pathways, PANoptosis was initially thought to be a combination of pyroptosis, apoptosis, and necroptosis, or crosstalk between these pathways. However, there is now extensive evidence that PANoptosis is a distinct cell death pathway [12,62]. PANoptosis cannot be blocked by ablation of molecular components from apoptosis, pyroptosis, or necroptosis alone [51,53]; instead, essential PANoptosome components (e.g., sensors, caspases, and/or RIPKs) are genetically required for PANoptosis. Thus, PANoptosis represents a distinct pathway at the cellular level.

The physiological roles of innate immune cell death (i.e., pyroptosis and PANoptosis) in cancers are complex, likely due to the intricate relationship between cancer development and inflammation induced by innate immune cell death. For example, genetic deletion of IRF1, a key transcription factor required to induce PANoptosis, promoted colorectal cancer in a mouse model [54], suggesting that cell death can prevent tumor development in this context. In contrast, both GSDMD and GSDME, which are key executioners in innate immune cell death, were suggested to promote cancer progression in HCC [64,65]. Similarly, IL-1β produced downstream of inflammasome activation is often pro-tumorigenic [66–68]. These anti- and pro-tumorigenic roles highlight that an in-depth understanding of the specific tumor and immune microenvironment will inform the use of innate immune cell death mechanisms in cancer treatment.

### Cell death pathways independent of caspase modulation

Beyond the pathways that are dependent on the activation or inhibition of caspases, emerging evidence has identified that several other cell death modalities operate independently of caspase activation status. While this was originally surprising in the field, given the widely accepted role of caspases as master regulators of cell death, the independence of these pathways from caspase regulation constitutes the major mechanistic difference from the pathways discussed in the previous section; many of the downstream effects, including the release of inflammatory mediators, are shared. In this category of cell death pathways, instead of engaging caspase machinery, these pathways often involve the disruption of essential cellular processes, such as energy metabolism and redox status, to drive the cell death. Key examples include ferroptosis, disulfidptosis, cuproptosis, and mitoxyperilysis.

Ferroptosis is driven by excessive iron-dependent peroxidation of polyunsaturated fatty acids [69,70]. First identified in cancer cells upon erastin treatment, ferroptotic cell death is blocked by iron chelation and lipophilic antioxidants [69]. Mechanistically, system Xc^−^ [SLC7A11/SLC3A2 (solute carrier family 7 member 11/family 3 member 2)] imports cystine to maintain glutathione (GSH) levels, which enables GPX4 (glutathione peroxidase 4) to reduce toxic lipid hydroperoxides into nontoxic lipid alcohols. Inhibition of either SLC7A11 (to reduce cystine uptake) or GPX4 (to block reduction of lipid peroxides) is therefore sufficient to trigger ferroptosis (Fig. 2) [69,71]. In addition, lipid remodeling enzymes, including ACSL4 (acyl-coenzyme A synthetase long-chain family member 4) and LPCAT3 (lysophosphatidylcholine acyltransferase 3), are also critical for ferroptosis induction [72]. Cancer cells exhibit differential sensitivity to ferroptosis, where many cancer cells avoid ferroptosis by enhancing antioxidant systems, such as through stabilization or up-regulation of GPX4 [73], SLC7A11 [74], or FSP1 (ferroptosis suppressor protein 1) [75], suggesting a cancer suppressive role for this pathway. Other redox-regulating enzymes [e.g., dihydroorotate dehydrogenase (DHODH) [76]] and ferritin degradation [77] also regulate cell sensitivity to ferroptosis. Crosstalk between caspase-dependent pathways and ferroptosis can also occur. For instance, the apoptosis inducer p53 also inhibits cystine uptake, which can sensitize cells to ferroptosis [74]. Inhibition of ferroptosis by *Acsl4* knockout can sensitize cells to necroptosis, while, conversely, ablation of MLKL or inactivation of RIPK3 can sensitize cells to ferroptosis [78,79].

**Fig. 2.**
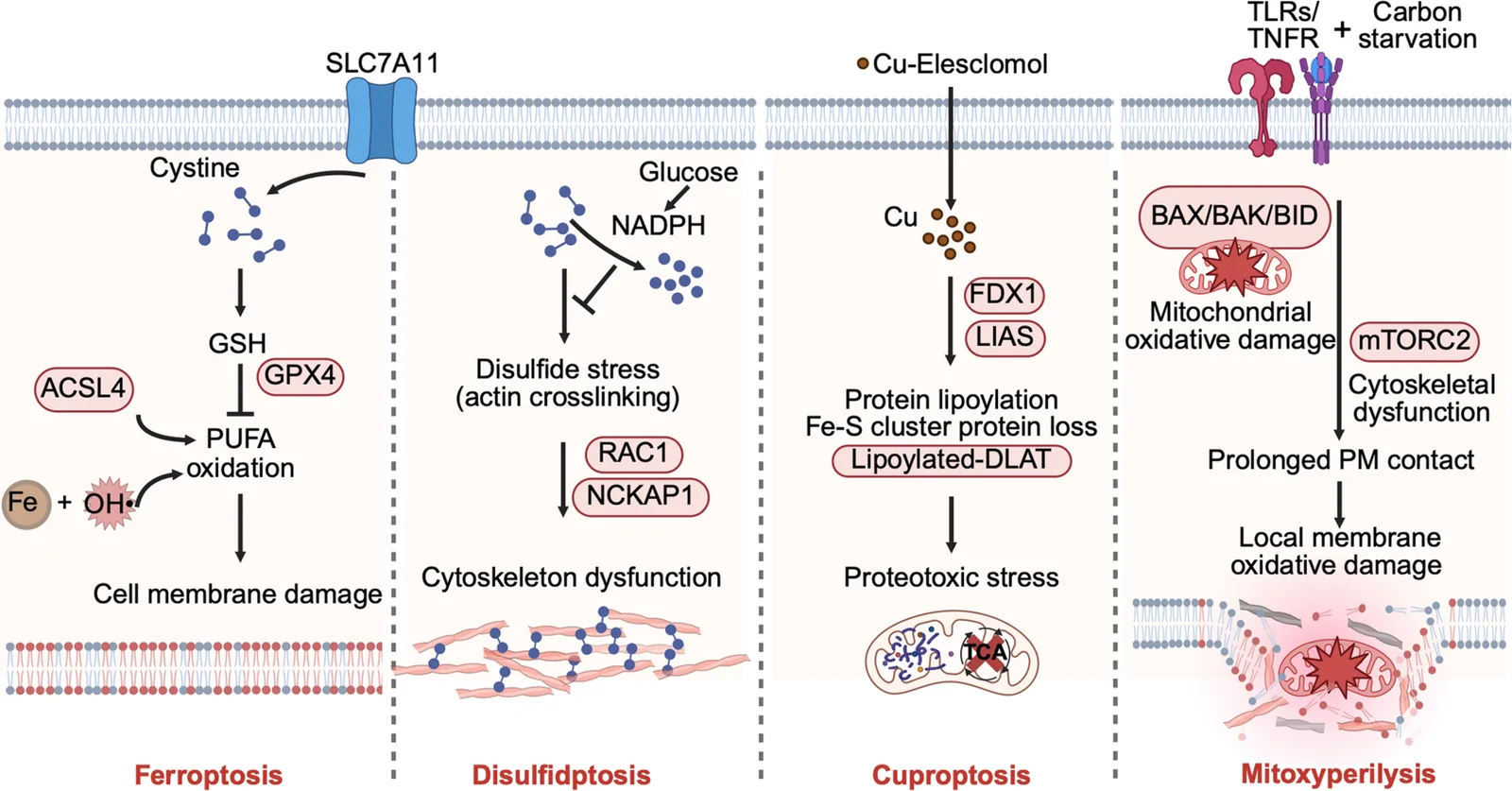
Ferroptosis, disulfidptosis, cuproptosis, and mitoxyperilysis—cell death pathways independent of caspase modulation. Ferroptosis is driven by iron-mediated PUFA peroxidation, leading to membrane damage. GPX4 mediates the detoxification of lipid hydroperoxides, and ACSL4 sensitizes cells to ferroptosis by enriching PUFA in cellular membranes. Disulfidptosis is caused by disulfide stress that leads to cytoskeleton disulfide crosslinking. RAC1 and NCKAP1 sensitize cells to disulfidptosis by promoting branched actin network formation in lamellipodia. Cuproptosis is induced by copper overload and the subsequent protein lipoylation-driven TCA cycle disruption and proteotoxic stress. FDX1 and LIAS are the key proteins involved in lipoylation of mitochondrial proteins, such as DLAT. Mitoxyperilysis is induced by the combination of innate immune activation (through TLR or TNFR engagement) and nutrient deprivation (i.e., carbon starvation). It is driven by BAX/BAK/BID-mediated mitochondrial dysfunction, leading to mTORC2-dependent, prolonged contact between the PM and oxidatively damaged mitochondria. This causes local oxidative damage at the PM, ultimately leading to membrane rupture. This figure was generated using BioRender. ACSL4, acyl-coenzyme A synthetase long-chain family member 4; BAK, B cell lymphoma 2 antagonist/killer 1; BAX, B cell lymphoma 2-associated X protein; BID, BH3-interacting domain death agonist; Cu, copper; DLAT, dihydrolipoamide S-acetyltransferase; FDX1, ferredoxin 1; Fe, iron; Fe–S, iron–sulfur; GPX4, glutathione peroxidase 4; GSH, reduced glutathione; LIAS, lipoic acid synthetase; mTORC2, mechanistic target of rapamycin complex 2; NADPH, reduced nicotinamide adenine dinucleotide phosphate; NCKAP1, NCK-associated protein 1; OH•, hydroxyl radical; PM, plasma membrane; PUFA, polyunsaturated fatty acid; RAC1, Ras-related C3 botulinum toxin substrate 1; SLC7A11, solute carrier family 7 member 11; TCA, tricarboxylic acid cycle; TLRs, Toll-like receptors; TNFR, tumor necrosis factor receptor.

Additionally, it is well-known that cancer cells rewire metabolic programs to meet their growth demands [1]. Therefore, metabolic perturbations can also give rise to distinct forms of cell death, with one example being disulfidptosis. Disulfidptosis is disulfide stress-driven cell death that occurs in cancer cells expressing high levels of SLC7A11 (SLC7A11^high^) under glucose starvation; in these cells, aberrant disulfide bonds cause cytoskeleton collapse, likely contributed by RAC1 and NCKAP1, and cell death (Fig. 2) [80]. Mechanistically, SLC7A11^high^ cancer cells rely on NADPH (reduced nicotinamide adenine dinucleotide phosphate) derived from glucose metabolism to reduce cystine [81]. Glucose starvation leads to excessive accumulation of cystine (due to high SLC7A11 activity), which causes disulfide stress, depletion of the NADPH pool, and aberrant disulfide crosslinking of actin to cause cell death [80]. This represents a metabolic vulnerability of SLC7A11^high^ cancer cells to cell death by glucose starvation [82]. Beyond its direct impact on cancer cells, disulfidptosis in tumor-associated T cells could also impact tumor burden [83].

Similar to how ferroptosis was identified by using chemical compounds to induce cell death, a study using the copper ionophore elesclomol identified a distinct copper-dependent form of cell death termed cuproptosis [84]. Copper binds to lipoylated mitochondrial tricarboxylic acid cycle (TCA) proteins [such as lipoylated dihydrolipoamide S-acetyltransferase (DLAT)], leading to loss of Fe–S cluster proteins, proteotoxic stress, and cell death (Fig. 2) [84]. Key regulators of cuproptosis include molecules involved in copper reduction [e.g., ferredoxin 1 (FDX1)] and protein lipoylation [such as lipoic acid synthetase (LIAS) and lipoyltransferase 1 (LIPT1)] [84]. Cancers with high mitochondrial oxidative metabolism and lipoylation activity may be more susceptible to cuproptosis [85], suggesting a context-specific effect on cancer cells.

Another cell death modality associated with metabolic disruption is the recently identified mitoxyperilysis. This pathway is uniquely positioned at the intersection of innate immune activation and metabolic disruption (e.g., carbon starvation or nutrient depletion), conditions that frequently co-occur in the TME. Under these conditions, mitochondria undergo oxidative stress and remain peripherally distributed, inducing local oxidative damage at the plasma membrane; this process is referred to as mitoxyperiosis [86]. Similar to other cell death pathways, mitoxyperilysis features the activation of caspases and lipid peroxidation; however, blocking caspase activity or lipid peroxidation does not block the cell death, demonstrating that mitoxyperilysis is a unique cell death pathway [86]. Instead, mechanistically, BAX, BAK, and BID mediate mitochondrial oxidative stress, while mTORC2 (mechanistic target of rapamycin complex 2) sustains prolonged mitochondria–plasma membrane contacts by suppressing cytoskeletal activity (Fig. 2) [86]. While this pathway was only identified recently, the initial studies have shown that cancer cells, which harbor intrinsically high oxidative stress levels [87] and mTOR (mechanistic target of rapamycin) activation [88], are particularly susceptible to this cell death modality [86]. Innate immune activation through pro-inflammatory cytokines such as TNF (tumor necrosis factor) sensitizes cancer cells to mitoxyperilysis, which has particular relevance in therapeutic strategies where TLR (Toll-like receptor) ligands are administered in vivo [86,89]. For example, intratumoral delivery of LPS (lipopolysaccharide) during fasting drove tumor regression in a B16 melanoma mouse model [86]. Historically, innate immune activation [90] and metabolic disruption [91] have existed as cancer therapies independently, supporting the possible cancer cell susceptibility to mitoxyperilysis and the feasibility of combination therapy. At this stage, mitoxyperilysis remains a newly described cell death pathway, and future investigations are needed in human cancer models and patient samples to further understand its physiological relevance in cancer; however, the preliminary evidence suggests the utility of targeting it to induce cancer cell death.

## Leveraging Cell Death Mechanisms in Cancer Treatment

Many available cancer treatments, including chemotherapies, radiation therapies, and biologics, induce cancer cell death via different mechanisms (Table 1). The selection of a particular mechanism to target is critical for improved precision and efficacy.

### Apoptosis, necroptosis, pyroptosis, and PANoptosis in cancer treatments

Apoptosis is a commonly leveraged modality in cancer treatment; historically, the effects of many chemotherapies and radiotherapies were attributed to apoptosis [5]. Cancer cells frequently overexpress anti-apoptotic proteins, most notably the BCL-2 family proteins (BCL-2, BCL-xL, MCL-1) that prevent mitochondrial release of pro-apoptotic factors and allow the cells to evade intrinsic apoptosis [92]. Similarly, death receptors required for extrinsic apoptosis (such as Fas/CD95) are frequently down-regulated in certain cancers [29]. The only current U.S. Food and Drug Administration (FDA)-approved cancer therapy that directly targets cell death molecules is venetoclax (approved in relapsed/refractory chronic lymphocytic leukemia, small lymphocytic lymphoma, or as part of combination therapy in acute myeloid leukemia), which targets BCL-2 family proteins to re-sensitize cancer cells to cell death [93–95]. In addition, radiation and many chemotherapeutic agents can up-regulate the expression of death receptors to re-sensitize cells to apoptosis [96,97], and there is substantial interest in developing IAP inhibitors or SMAC mimetics to do the same [98]. When developing such therapies to induce apoptosis, the impact on the TME should be considered. For instance, macrophage efferocytosis of apoptotic cells drives an immunosuppressive macrophage phenotype that can limit CD8^+^ T cell infiltration and promote pancreatic cancer metastasis [99]. The negative membrane charge of apoptotic cells can also induce sprouting of endothelial cells that impacts angiogenesis and vascular remodeling in tumors [100]. Furthermore, a recent study showed that staurosporine, a canonical apoptosis-inducing agent, can also induce lytic cell death, PANoptosis [101]. This finding suggests the potential for classical apoptosis inducers to drive immunogenic cell death that can be a double-edged sword to either stimulate immune activation or drive tolerance in the TME, depending on the tumor type. These findings highlight the need to carefully re-evaluate the role of apoptosis in driving cancer therapy effects based on recent advances in our understanding of different cell death pathways.

Currently, there are no FDA-approved anticancer therapies that specifically induce necroptosis, pyroptosis, or PANoptosis; however, preclinical studies support therapeutic targeting of these cell death pathways for cancer treatment (Table 1). In a mouse model, the specific induction of intratumoral necroptosis was shown to activate a BATF3^+^ (basic leucine zipper ATF-like transcription factor 3) cDC1-dependent antitumor CD8^+^ T cell response, which led to tumor regression [102]. Pan-caspase inhibitor (e.g., zVAD) treatment, which induces necroptosis, has also been tested in combination with chemoradiotherapy; this treatment increased immunogenicity and antitumor immune responses in mice [103]. Furthermore, the immunogenicity of cell death could extend beyond the cell death event itself, which should be considered when designing treatments. For instance, necroptotic cells can still transcribe inflammatory cytokines [104], and nuclear factor κB (NF-κB) signaling in necroptotic cells was critical for priming tumor antigen-specific CD8^+^ T cells for antitumor immune responses [105]. The key effector kinase RIPK3, which is involved in necroptosis and PANoptosis, can be silenced by transcriptional start site methylation in human cancers, suggesting that using hypomethylating agents to re-express RIPK3 may sensitize cancers to necroptosis [37]. However, the inflammatory nature of necroptosis can also be a double-edged sword, leading to immunosuppression, such as during pancreatic oncogenesis [39] and in breast tumors [40]. These findings highlight that cellular context, disease context, and inflammatory milieu can influence whether necroptotic signaling exerts tumor-promoting or tumor-restraining functions. Thus, disease context is critical when considering the therapeutic targeting of necroptosis. With these caveats in mind, the proinflammatory nature of these pathways makes them particularly enticing therapeutic targets, especially considering the priming effect for adaptive anticancer immune responses.

Promoting innate immune cell death has also shown preclinical promise. Targeting the executioner phase is one such strategy. For instance, a small molecule GSDMD agonist has shown promising antitumor effects in animal models [106]. Similarly, while the loss of GSDME function in tumor cells promoted tumor growth, GSDME ectopic expression in GSDME-deficient tumors inhibited tumor growth; both effects are T cell-dependent, highlighting the innate immune cell death potency in regulating adaptive anticancer responses [107]. Cleavage of other gasdermins has also been demonstrated in cancer cell death under other contexts, including granzyme A cleavage of GSDMB [108] and caspase-8-induced GSDMC activation [109]. These studies highlight a tumor-suppressive role of gasdermins. Of note, GSDME expression is silenced in many human cancers [110]. Therefore, strategies to induce GSDME to drive cell death for cancer treatment should consider both the re-expression of GSDME as well as its activation to effectively induce cell death. These preclinical tests require extensive in vivo and clinical ex vivo validation to begin the process of translation. Beyond targeting the executioner level, upstream induction of PANoptosis has also been tested as an anticancer strategy preclinically. Combined treatment with an oral nuclear export inhibitor and intratumoral injection with interferon drives ZBP1 (Z-DNA-binding protein 1)-dependent PANoptosis and decreases tumor burden in a mouse model [55]. Additionally, PANoptosis induced by cytokine combinations [TNF plus interferon-γ (IFN-γ)] has demonstrated efficacy in inducing cell death across several cancer lineages and in vivo [56]. A possible advantage of PANoptosis induction in cancer treatment is the potential to overcome cancer cell resistance that might develop to apoptosis, necroptosis, or pyroptosis; molecular components associated with these pathways are activated in PANoptosis, but their inhibition does not prevent PANoptotic cell death [12]. Therefore, activating PANoptosis could still induce cancer cell death when critical components of the apoptosis, pyroptosis, or necroptosis pathways have been silenced during cancer development [37,56,110].

While each of these cell death modalities holds strong potential to induce anticancer immune responses in vivo, the pro-inflammatory nature of innate immune cell death, specifically pyroptosis and PANoptosis through production of IL-1β, can also induce immunosuppression [111] and has been associated with cancer growth in some contexts [112]. For example, an inflammatory TME can favor the accumulation of myeloid-derived suppressor cells (MDSCs) that suppress T cell activity, enhance angiogenesis, and promote metastasis [113]. In pancreatic ductal adenocarcinoma, IL-1 signaling drives fibroblasts toward an inflammatory phenotype and promotes cancer progression [114]. Systemic inflammation can also trigger up-regulation of adhesion molecules by endothelial cells to promote tumor cell vascular adhesion and metastasis [115]. Thus, the therapeutic value of activating inflammatory cell death is highly context-dependent in cancer. As such, disease stage, inflammatory microenvironment, cell death molecule expression profiles, and effects in both cancer and stromal cells should be taken into account when designing cell death-targeting therapeutic approaches. By considering these factors, there is great potential to develop effective therapeutics.

### Emerging strategies to induce ferroptosis, disulfidptosis, cuproptosis, and mitoxyperilysis

The characterization of distinct cell death modalities opens the possibility for inducing cancer cell death through unique approaches. Similar to necroptosis, pyroptosis, and PANoptosis, there are currently no FDA-approved treatments to activate ferroptosis, disulfidptosis, cuproptosis, and mitoxyperilysis, but preclinical data suggest that these could be promising strategies when used appropriately [116] (Table 1).

Many oncogenic programs in cancer cells cause increased resistance to ferroptosis, for example, SLC7A11, which inhibits ferroptosis, is up-regulated during RAS-mediated transformation and is required for oncogenic KRAS-driven tumor growth [117], suggesting that this pathway could be therapeutically targeted to modulate cancer cell susceptibility to ferroptosis. Some cancer treatments already in use impact cancer cell ferroptosis. For instance, immune checkpoint blockade treatment activated T cells to enhance ferroptosis-related lipid peroxidation in cancer cells, thereby contributing to the antitumor efficacy of immunotherapy [118]. Induction of ferroptosis is particularly of interest for killing drug-resistant cells, as these cells acquire sensitivity to GPX4 inhibition [119,120]. However, using ferroptosis-inducing agents as tumor therapy will require careful consideration to mitigate systemic toxicity, delivery challenges (e.g., poor stability and water solubility of current drugs), and side effects [121]. Selective tumor delivery of ferroptosis inducers could address these challenges, using strategies such as nanoparticle-based delivery systems [121,122], although this remains an active area of investigation. In addition, ferroptosis can also favor tumor growth and impede antitumor immune responses. For example, ferroptosis of tumor neutrophils was shown to cause an immunosuppressive microenvironment that drives tumor growth [123]. Moreover, pancreatic cancer cells undergoing ferroptosis as a result of GPX4 inhibition can secrete immunosuppressive metabolites that inhibit T cell function and antitumor responses [124]. Therefore, ferroptosis induction in combination with other therapeutic treatments such as immune checkpoint blockade [125] could represent an improved strategy for anticancer therapy.

As mentioned above, to evade ferroptosis, many cancer cells up-regulate SLC7A11 [126]. This up-regulation confers potential disulfidptosis sensitivity. The induction of disulfidptosis with a glucose transporter inhibitor decreases SLC7A11^high^ tumor volume in mouse models [80], suggesting that disulfidptosis can drive an antitumor effect. On the other hand, disulfidptosis may also occur in tumor-infiltrating CD8^+^ T cells, contributing to their exhaustion and therefore promoting tumor growth [83]. A recent effort using nanoparticles containing glucose oxidase to trigger disulfidptosis, along with other lytic forms of cell death, showed a promising effect in stimulating antitumor immunity [127]. While SLC7A11^low^ cancer cells are resistant to disulfidptosis, they are sensitive to ferroptosis [128]. Therefore, a therapeutic strategy to induce both ferroptosis and disulfidptosis may be effective against broader cancer subtypes.

Apart from ferroptosis and disulfidptosis, recent studies suggest a role for cuproptosis in specific cancers. For example, METTL16-driven regulation of FDX1 and DLAT lipoylation modulated cuproptosis sensitivity in gastric cancer models [129], and cuproptosis regulators correlated with disease phenotypes in HCC tissues and models [130]. Therapeutically, an engineered nanoplatform based on Cu_9_S_8_ can increase copper ion levels in tumor cells, thereby sensitizing tumor cells to cuproptosis [131]. Clinically, copper-modulating therapies have a longer history than cuproptosis itself. For example, clinical trials of the copper ionophore elesclomol conducted prior to the establishment of cuproptosis as a unique cell death pathway found no beneficial effect in melanoma [84,132]. Yet, a recent study identified a specific vulnerability of GNAQ/11 (guanine nucleotide-binding protein subunit α q/11)-mutant uveal melanoma to elesclomol [133], suggesting a mutation-specific benefit to inducing cuproptosis that has yet to be clinically tested. Conversely, copper chelation with tetrathiomolybdate showed no clinical efficacy in treating hormone-refractory prostate cancer [134], while tetrathiomolybdate has been associated with a survival benefit in high-risk triple negative breast cancer patients [135]. Therefore, copper metabolism and associated cuproptosis are likely to have context-dependent roles in cancer biology and tumor therapy.

Finally, recent evidence has shown mitoxyperilysis to be a promising strategy to induce anticancer cell death. Mitoxyperilysis occurs at the intersection of innate immunity and metabolism [86]. In the context of tumor therapy, innate immune activation by intratumoral injection of bacterial lysate was investigated historically by bone sarcoma surgeon William B. Coley in the 19th century [136]. These “Coley’s toxins” successfully treated many patients with inoperable bone and soft-tissue sarcomas but came under great criticism, and their use was discontinued [136]. Targeting metabolism is also a tested strategy for cancer treatment [137]. In modern practice, fasting schemes to block or slow cancer growth have been employed, but the efficacy of fasting alone is limited [91,138]. Thus, inducing mitoxyperilysis through a combination treatment strategy, using innate immune signals from TLRs or the TNF receptor alongside metabolic disruption, holds therapeutic promise in cancer [86]. In a mouse subcutaneous tumor model, either intratumoral injection of the TLR ligand, LPS, or fasting alone was tumor static, halting tumor growth [86]. Combined treatment of intratumoral LPS with fasting was tumoricidal, inducing robust mTOR-dependent tumor necrosis with key features of mitoxyperilysis such as peripherally localized damaged mitochondria in necrotic tumor cells [86]. While some tumor cells have limited expression of TLRs [89], induction of mitoxyperilysis can still occur through TNF, as the nearby immune cells will produce TNF in response to TLR ligand administration in vivo. In fact, many TLR agonists are being evaluated in clinical trials for solid tumor treatment, but these have been associated with side effects consistent with systemic activation of the innate immune system [90]. Therefore, targeted delivery of TLR agonists, such as through intratumoral injection, may improve clinical benefit. Short-term fasting induces the production of ketone bodies from the liver, which can be utilized as an energy source for many normal tissues, but not by most cancer cells [139]. Thus, nutrient deprivation may create a therapeutic window to favor the induction of mitoxyperilysis in tumors but not in normal tissues. Therefore, integrating fasting schemes (e.g., calorie restriction, intermittent fasting, and ketogenic diet) into the current trials testing TLR ligands in solid tumor treatment holds strong promise for mitoxyperilysis-based anticancer therapy. Such a strategy would need to consider fasting scheme and dosing to optimize therapeutic benefit while limiting toxicity and side effects, highlighting potential clinical complexity. In addition, future studies are needed to define strategies that enhance mitoxyperilysis in tumor cells while sparing nontumor tissues. Identification of predictive biomarkers may be helpful to guide patient stratification, allowing for optimization of treatment schemes to minimize side effects.

## The Outlook of Cell Death Induction in Cancer Treatment

The induction of cell death in cancer remains a central treatment strategy; yet, challenges remain. Despite advances in mouse modeling, there remains a critical lack of appropriate preclinical models for studying many cancer types and their immunogenicity. Several models are based on patient-derived xenografts, which require the use of immunodeficient mice. Neither conventional tumor cell assays nor immunodeficient xenografts can fully capture tumor heterogeneity, stromal regulation, or immune-mediated therapy effects. This limits the ability to assess therapeutics that target cell death for their effects on the broader immune response to fully understand their safety and efficacy. Continued development of optimal genetic mouse models that mimic human cancers will be critical for target identification and validation, and to appropriately assess therapeutics.

Additionally, it can be difficult to define the in vivo relevance of specific cell death pathways. Many current assays measure general tissue damage rather than pathway-specific features. Furthermore, there is extensive molecular overlap between some cell death pathways, as well as a lack of specific markers. For example, detection of cleaved caspase-3 in tumor tissue may suggest that apoptosis is occurring, but caspase-3 can also be activated during PANoptosis [57,140]. Similarly, lipid peroxidation [141], disulfide formation [142], and mitochondrial damage [143] are part of a general oxidative stress response and cannot be used to distinguish among the cell death pathways. Therefore, rigorous evaluation of a panel of parameters while examining cell death in vivo is necessary to define its physiologic relevance. Furthermore, the intrinsic plasticity and replication of cancer cells lead to the acquisition of resistance mutations that can circumvent a targeted pathway [144]. Thus, understanding and engaging multiple cell death pathways, or pathways such as PANoptosis that engage multiple components, can be guided by the cancer cell’s gene expression and metabolic profile to provide more effective therapeutics. To do this, identifying specific markers that define a cancer cell’s susceptibility to particular cell death pathways will allow for improved biomarker-guided identification of cancers by their sensitivity to certain cell death pathways, to match the right therapeutic strategy to each patient. Candidate markers include pathway-specific features linked to cell death pathways (Table 2), as well as immune state markers (e.g., DAMP release, inflammatory cytokines, type I interferon signatures, antigen-presenting cell activation markers, and T cell infiltration markers).

When considering therapeutic targets, targeting the upstream sensor molecule(s) may be the best approach, as this would have the most direct effect by globally limiting downstream pathway activation. Other potential targets could be downstream molecules, such as the effectors (e.g., caspases and RIPKs), gasdermins and pore-formation, and plasma membrane rupture (PMR) steps in cell death. PMR is driven by molecules such as NINJ1 (ninjurin 1) [145] and SIGLEC12 (sialic acid binding Ig like lectin 12) [146]. Given the critical role of PMR in the release of immunostimulatory DAMPs, targeting this event could represent a way to modulate the inflammation associated with cell death induction in cancers.

Finally, comprehensively understanding the double-edged nature of cancer cell death will be critical to the design of therapeutic approaches. For instance, many inflammatory cell death pathways can be immunosuppressive in vivo [39,66]. Additional mechanistic studies are needed to define how different forms of tumor cell death could remodel the TME, and how this impacts immunogenicity depending on pathologic context. The goal would be to selectively block immunosuppression while allowing for cancer cell death, to maximize immune sensitization. Furthermore, tumor necrosis has long been associated with poor prognosis and metastasis risk [40,147], potentially due to chronic tissue inflammation associated with cell death. Therefore, while inducing cancer cell death represents a promising treatment strategy for some cancers, cell death-related inflammation could also contribute to negative outcomes in some contexts [40,148], requiring careful mitigation. Identifying which specific cell death modalities drive this risk, paired with an understanding of cancer-specific consequences of inflammation, will be key in developing cancer therapeutics [149]. Considering all these points, the induction of cancer cell death in specific, targeted contexts, with or without immune checkpoint blockade, represents a powerful approach to improve therapeutic efficacy.

Overall, the mechanistic understanding of cell death modalities and their innate immune, inflammatory consequences represents a critical foundation with profound fundamental and therapeutic implications. Therapeutically targeting cell death pathways remains a viable strategy that has shown great promise in preclinical and clinical settings, making it worthy of future investment and research. Ultimately, harnessing the full power of innate immunity and regulated cell death, alone and in combination with immune checkpoint blockade and to overcome cancer cell resistance mechanisms, holds transformative potential for next-generation cancer therapy.
